# A219 EARLY CANADIAN EXPERIENCE WITH BALLOON CRYOTHERAPY FOR BARRETT’S ESOPHAGUS

**DOI:** 10.1093/jcag/gwad061.219

**Published:** 2024-02-14

**Authors:** R Sullivan, A Fazal, M Gupta, P Belletrutti, C Wong

**Affiliations:** University of Alberta, Edmonton, AB, Canada; University of Alberta, Edmonton, AB, Canada; University of Calgary, Calgary, AB, Canada; University of Calgary, Calgary, AB, Canada; University of Alberta, Edmonton, AB, Canada

## Abstract

**Background:**

Balloon Cryotherapy (bCr) is a novel endoscopic treatment modality for Barrett’s esophagus (BE) associated dysplasia. Cryotherapy offers an additional approach, particularly in cases refractory to or with an anatomical impediment to radiofrequency ablation (RFA), currently first line in endoscopic eradiation therapy (EET). The use of bCr is new in Canada and it was introduced in Alberta in February 2023.

**Aims:**

To present the early experience of bCr use for BE from a provincial Canadian cohort.

**Methods:**

An Alberta health technology assessment approved bCr, which was then incorporated into the provincial BE treatment algorithm. Indications for bCr include: 1) RFA-refractory, 2) anatomical impediment to RFA, 3) need for concomitant biopsy/EMR, and 4) pain with RFA. All patients who underwent bCr for BE in Alberta from February to September 2023 at two tertiary care centers were included. Data on demographics, BE lesion characteristics, prior BE treatments, and bCr use are presented.

**Results:**

During the 8-month period, 22 patients underwent a total of 32 bCr treatments. The median age was 68, with 77% of patients being male. At baseline, 81% had long-segment BE (≥3 cm), with a median maximal BE length of 7cm. The majority had baseline histology of high-grade dysplasia (HGD) (n=11, 50%), followed by low-grade dysplasia (LGD) (n=5, 22%), T1a (n=4, 18%), T1b (n=1, 4.5%), and intestinal metaplasia (IM) (n=1, 4.5%). All patients had previously received EET, with 77% undergoing a combination of EMR, RFA, APC, and/or ESD. Most had received RFA (n=18, 81%, median sessions=5) and EMR (n=13, 59%, median sessions=2). Some had prior APC (n=10, 45%, median sessions=1) and one had ESD. Prior to bCr, most patients had converted to short-segment (ampersand:003C3cm) BE (n=16, 72%), and histology was predominately IM (31%), followed by visible BE though no biopsy (27%), HGD (22%), T1a (13%), and LGD (4.5%). The primary indication for bCr was RFA-refractory BE (n=17, 77%), followed by anatomical impediment to RFA (n=4, 18%), and concomitant EMR (n=1, 4%). No procedures were aborted due to technical difficulties and there were no major adverse events such as perforation, bleeding, or pain requiring hospitalization. Among those with follow-up after a bCr session (n=19, 59%), one reported pain, two reported self-limited dysphagia, and one had a stricture requiring dilation. Of the 7 patients with post-bCr endoscopy, all had no dysplasia on the first set of biopsies (no IM, n=4; IM, n=2; indefinite for dysplasia, n=1).

**Conclusions:**

Our early experience with bCr demonstrates there is a need for additional EET for BE. Cryotherapy, in accordance with gastroenterology society guidelines, is a promising next step in the BE algorithm. In our provincial cohort, bCr has shown technical success, and preliminary data suggests that it is safe and effective in ablating resistant BE segments.

**Funding Agencies:**

None

